# Phenylbutazone induces expression of MBNL1 and suppresses formation of MBNL1-CUG RNA foci in a mouse model of myotonic dystrophy

**DOI:** 10.1038/srep25317

**Published:** 2016-04-29

**Authors:** Guiying Chen, Akio Masuda, Hiroyuki Konishi, Bisei Ohkawara, Mikako Ito, Masanobu Kinoshita, Hiroshi Kiyama, Tohru Matsuura, Kinji Ohno

**Affiliations:** 1 Division of Neurogenetics, Center for Neurological Diseases and Cancer, Nagoya University Graduate School of Medicine, Nagoya, Japan; 2 Division of Functional Anatomy and Neuroscience, Nagoya University Graduate School of Medicine, Nagoya, Japan; 3 Department of Frontier Health Sciences, Graduate School of Human Health Sciences, Tokyo Metropolitan University, Tokyo, Japan; 4 Division of Neurology, Department of Medicine, Jichi Medical University, Shimotsuke, Japan

## Abstract

Myotonic dystrophy type 1 (DM1) is caused by abnormal expansion of CTG repeats in the 3′ untranslated region of the *DMPK* gene. Expanded CTG repeats are transcribed into RNA and make an aggregate with a splicing regulator, MBNL1, in the nucleus, which is called the nuclear foci. The nuclear foci sequestrates and downregulates availability of MBNL1. Symptomatic treatments are available for DM1, but no rational therapy is available. In this study, we found that a nonsteroidal anti-inflammatory drug (NSAID), phenylbutazone (PBZ), upregulated the expression of MBNL1 in C2C12 myoblasts as well as in the *HSA*^LR^ mouse model for DM1. In the DM1 mice model, PBZ ameliorated aberrant splicing of *Clcn1, Nfix*, and *Rpn2*. PBZ increased expression of skeletal muscle chloride channel, decreased abnormal central nuclei of muscle fibers, and improved wheel-running activity in *HSA*^LR^ mice. We found that the effect of PBZ was conferred by two distinct mechanisms. First, PBZ suppressed methylation of an enhancer region in *Mbnl1* intron 1, and enhanced transcription of *Mbnl1* mRNA. Second, PBZ attenuated binding of MBNL1 to abnormally expanded CUG repeats *in cellulo* and *in vitro*. Our studies suggest that PBZ is a potent therapeutic agent for DM1 that upregulates availability of MBNL1.

Myotonic dystrophy type 1 or dystrophia myotonica type 1 (DM1), is the most common form of adult-onset muscular dystrophy. The disease is dominantly inherited and affects multiple tissues, exhibiting symptoms including muscle hyperexcitability (myotonia), progressive muscle wasting, cardiac conduction defects, cardiomyopathy, insulin resistance, and neuropsychiatric disorders^1^. DM1 is caused by abnormally expanded CTG repeats in the 3′ untranslated region (3′ UTR) of the DM protein kinase gene (*DMPK*) on chromosome 19. Unaffected individuals have 5–30 copies of CTG repeats, whereas DM1 patients carry 50 to several thousand copies^2^,^3^. In DM1 patients, the transcribed expanded repeats make aggregates in the nucleus, which are recognized as distinct nuclear RNA foci^4^,^5^. An RNA-binding protein, the muscleblind-like protein 1 (MBNL1), is sequestered to the CUG repeats^6^,^7^, whereas another RNA-binding protein, the CUG-binding protein, Elav-like family member 1 (CELF1), is not sequestered but is activated in DM1 somehow by the abnormally expanded CUG repeats^8^. As sequestration of MBNL1 to RNA foci downregulates availability of MBNL1, upregulation of MBNL1 expression is expected to ameliorate toxicity of abnormally expanded CUG repeats^9^,^10^.

Using the drug repositioning strategy, in which a drug used for a specific disease is applied to another unrelated disease, we found that phenylbutazone (PBZ) upregulates the expression of MBNL1 in C2C12 mouse myoblasts. We identified, in the skeletal muscle of *HSA*^LR^ mice model for DM1, that PBZ reduces the number of central nuclei, partially normalizes aberrant splicing of *Clcn1* encoding the skeletal muscle chloride channel, and increases expression of the chloride channel. PBZ enhances transcription of *Mbnl1* mRNA, through demethylation of an enhancer region in *Mbnl1* intron 1. In addition, PBZ suppresses binding of MBNL1 to CUG RNA *in cellulo* as well as *in vitro*.

## Results

### Phenylbutazone (PBZ) elevates MBNL1 expression in myogenic C2C12 cells

NSAIDs are effective for neuromuscular and neurodegenerative disorders including myotonic dystrophy type 2^11^,^12^, Duchenne muscular dystrophy^13^, Alzheimer’s disease^14^,^15^, and Parkinson disease^16^. Since upregulation of MBNL1 is a promising therapeutic target of DM^17^, we screened NSAIDs for upregulation of MBNL1 using C2C12 mouse myoblasts. After culturing the cells with each drug at 50 μM for 24 h, we performed real-time RT-PCR analysis and found that PBZ promoted expression of *Mbnl1* mRNA 1.3-fold. We observed that PBZ upregulated *Mbnl1* mRNA in C2C12 cells in a dose-dependent manner up to 1.9-fold (Supplementary Fig. S1a).

**Figure 1:**
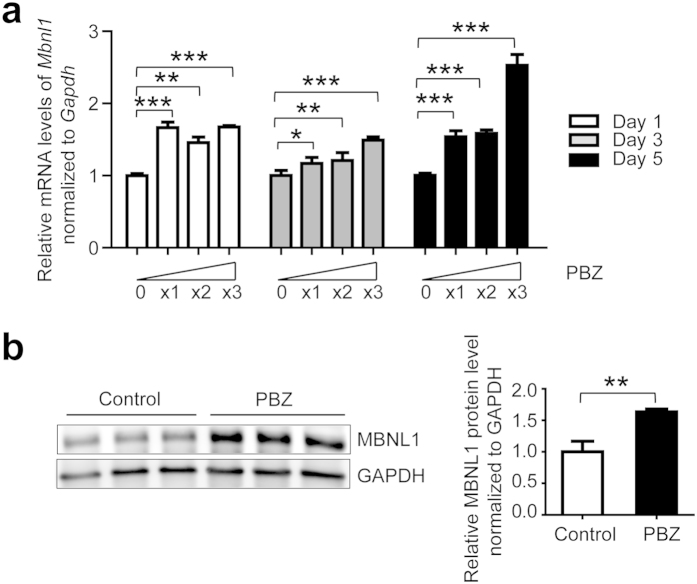
PBZ upregulates MBNL1 expression in C2C12 myoblasts and myotubes. (**a**) Real-time RT-PCR to estimate expression of *Mbnl1* during myogenic differentiation of C2C12 myoblasts. Cells were treated with variable concentrations of PBZ from differentiation day 0. Concentrations of PBZ: 0, 0 μM; x1, 324 μM (100 ng/μl); x2, 648 μM (200 ng/μl); and x3, 972 μM (300 ng/μl). Expression levels of *Mbnl1* are normalized to that of *Gapdh* and the relative mRNA expression levels are normalized to that of cells treated with 0 μM of PBZ on the same differentiation day. The relative expression levels of *Mbnl1* in untreated cells normalize for that on day 1 were 1.00 ± 0.03 on day 1, 1.56 ± 0.10 on day 3, and 1.21 ± 0.04 on day 5 (mean and SD, *n* = 3). The mean and SD of three independent experiments are indicated. The data are analyzed by two-way ANOVA followed by Bonferroni post-hoc test. *p < 0.05; **p < 0.01; ***p < 0.001. (**b**) Western blotting analysis of MBNL1 in differentiated C2C12 myotubes. Cells were treated with 324 μM (100 ng/μl) of PBZ from differentiation day 0, and whole cell lysates were extracted on day 5 after induction of differentiation. Left panel shows representative blots, and right panel shows quantitative analysis of signal intensities. Expression levels of MBNL1 protein in untreated cells (control) and treated cells PBZ (PBZ) are normalized to that of GAPDH and the relative protein expression levels are normalized to that of control. The mean and SD of three samples in each group are indicated in the right graph. The data were analyzed by Student’s *t*-test. **p < 0.01.

We next examined the effect of PBZ on MBNL1 expression during myogenic differentiation. We harvested total RNA from C2C12 cells on differentiation days 1, 3, and 5, and performed real-time RT-PCR analysis. Our analysis showed that PBZ upregulated *Mbnl1* mRNA in a dose-dependent manner on days 1, 3, and 5 (Fig. 1a and Supplementary Fig. S1b). Consistently, Western blot analysis showed that PBZ upregulated MBNL1 protein level in C2C12 cells on differentiation day 5 (Fig. 1b). These results suggest that PBZ enhances MBNL1 expression in myogenic cells in a dose-dependent manner.

### PBZ improves running wheel activity and muscle histopathology in *HSA* ^LR^ mice

**Figure 2:**
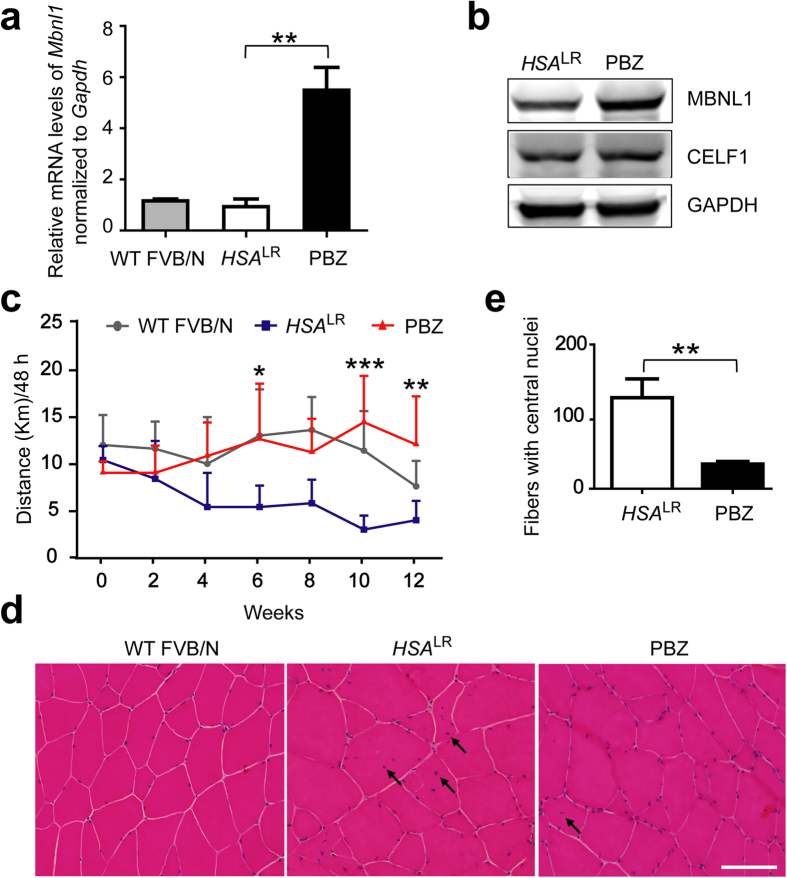
In *HSA*^LR^ mice, PBZ upregulates MBNL1 expression in skeletal muscle, and improves muscle weakness and histopathology. Three groups (*n* = 5 in each group) of mice were analyzed: (i) Untreated wild-type FVB/N mice (WT FVB/N); (ii) untreated *HSA*^LR^ mice (*HSA*^LR^); and (iii) PBZ-treated *HSA*^LR^ mice (PBZ). (**a**) Real-time RT-PCR analysis to estimate expression of *Mbnl1* in tibialis anterior (TA) muscles. Expression levels of *Mbnl1* are normalized to the level of *Gapdh* and relative mRNA expression levels are normalized to WT FVB/N. The mean and SD of five mice in each group are indicated. The data were analyzed by one-way ANOVA followed by Tukey’s test. **p < 0.01. (**b**) Western blotting analysis of MBNL1 in mouse TA muscles. Whole protein was extracted from TA muscles. A representative blot of three independent experiments is shown. (**c**) Voluntary movements quantified by a counter-equipped running wheel. Plots show mean and SD of the running distance over 48 h. The data were analyzed using two-way repeated measures ANOVA followed by Bonferroni post-hoc test. *p < 0.05, **p < 0.01, ***p < 0.001 compared to untreated *HSA*^LR^ mice. (**d**) Representative hematoxylin and eosin staining of quadriceps muscles in three groups of mice. Arrows point to central nuclei of muscle fibers. Scale bar = 300 μm. (**e**) The number of fibers with central nuclei was counted in eight randomly selected visual fields of a section of quadriceps muscle in untreated *HSA*^LR^ mice (*HSA*^LR^) or PBZ-treated *HSA*^LR^ mice (PBZ). The mean and SD of five mice in each group are indicated. The data were analyzed by unpaired Student’s *t*-test. **p < 0.01.

We next analyzed the effects of PBZ on *HSA*^LR^ mice, which carried 250 CUG repeats driven by a muscle-specific promoter. *HSA*^LR^ mouse is an established model for DM1^18^. PBZ (16.7 mg/kg/day) was given to the *HSA*^LR^ mice in drinking water for 12 weeks from age 8 weeks. We confirmed that the PBZ treatment did not affect the body weight (Supplementary Fig. S2a) and serum levels of liver function parameters (Supplementary Table S1) in *HSA*^LR^ mice. Consistent with the results of C2C12 cells, our analysis showed that PBZ elevated expression of *Mbnl1* mRNA and protein in tibialis anterior (TA) muscles (Fig. 2a,b) as well as quadriceps muscles (Supplementary Fig. S2b,S2c) of *HSA*^LR^ mice. We also found that PBZ treatment did not affect expression of CELF1 in these mice (Fig. 2).

We next analyzed the voluntary wheel running exercise and grip strength for these mice during PBZ treatment. We found that PBZ treatment increased the number of wheel rotations and grip strength in *HSA*^LR^ mice to a similar level of wild-type mice (Fig. 2c, Supplementary Fig. S2d), suggesting that PBZ ameliorates muscle weakness.

In *HSA*^LR^ mice, affected muscles show central nuclei, split fibers, ring fibers, and markedly increased fiber size variations^18^. We stained muscle sections of untreated wild type FVB/N mice, untreated *HSA*^LR^ mice, and PBZ-treated *HSA*^LR^ mice with hematoxylin and eosin (H&E), and found that PBZ significantly reduced the number of muscle fibers with central nuclei (Fig. 2d,e).

### PBZ corrects abnormal splicing of *Clcn1* gene and increases CLCN1 protein expression

**Figure 3:**
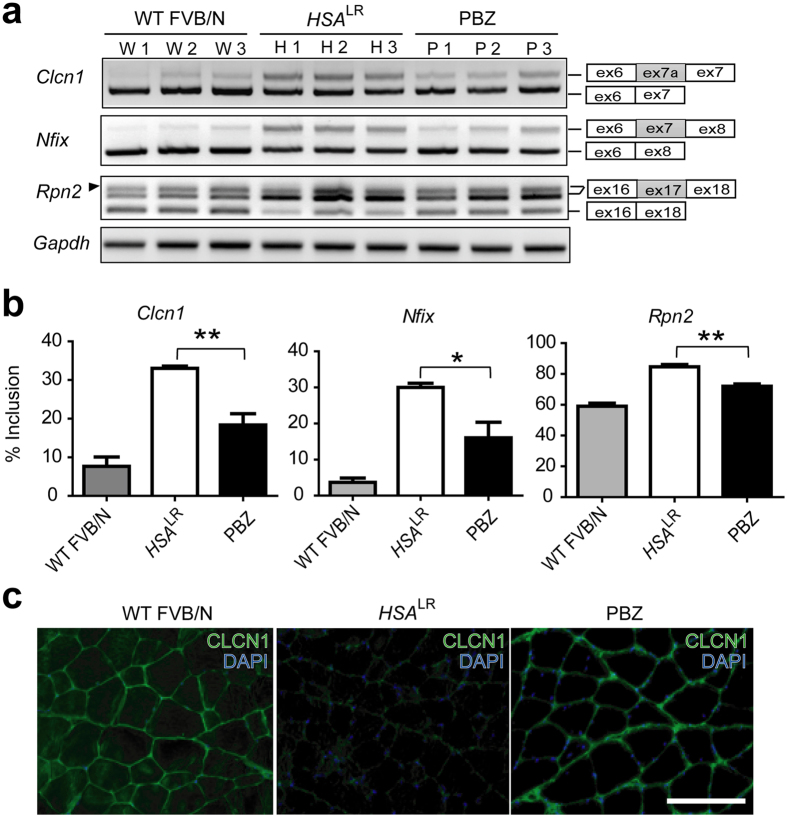
PBZ improves aberrant splicing of pre-mRNAs in *HSA*^LR^ mice. (**a**) RT-PCR analysis of splicing of *Clcn1* exon 7a, *Nfix* exon 7 and *Rpn2* exon 17 in TA muscles of untreated wild-type FVB/N mice (WT FVB/N), untreated *HSA*^LR^ mice (*HSA*^LR^) and PBZ-treated *HSA*^LR^ mice (PBZ). In *Rpn2*, two exon-included bands were detected. The minor upper band (arrowhead) was a consequence of alternative 5′ splice site selection, which contained 29 nt downstream of exon 17. The alternative *Rpn2* transcript is annotated as Mbnl1.jSep07 in the AceView gene database. RT-PCR of three mice in each group is indicated. (**b**) The ratio of inclusion of an exon was calculated as explained in Methods. The mean and SD of three mice are indicated. The data was analyzed by one-way ANOVA followed by Tukey’s test. *p < 0.05, **p < 0.01. (**c**) Representative immunofluorescent detection of CLCN1 protein (green) in quadriceps muscles of untreated wild-type FVB/N mice (WT-FVB/N), untreated *HSA*^LR^ mice (*HSA*^LR^) and PBZ-treated *HSA*^LR^ mice (PBZ). Nuclei were counterstained with DAPI (blue). Scale bar = 300 μm.

More than 100 aberrant splicing events are observed in the skeletal muscles of *HSA*^LR^ mice model^19^. Among them, abnormal alternative splicing of *Clcn1* results in a loss-of-function of the membrane-associated chloride channels and causes myotonia, which is one of the major symptoms of DM1. Since MBNL1 enhances skipping of *Clcn1* exon 7a in cultured cells^20^, we examined whether PBZ corrects the aberrant inclusion of this exon in *HSA*^LR^ mice using RT-PCR (Fig. 3a). Our analysis demonstrated that PBZ suppressed abnormal inclusion of exon 7a to 18%, compared to the 33% inclusion in untreated *HSA*^LR^ mice (Fig. 3b). We also found that PBZ ameliorated aberrant inclusions of *Nfix* exon 7 and *Rpn2* exon 17 in *HSA*^LR^ mice^19^. In contrast, PBZ did not affect splicing of these exons in wild-type FVB/N mice (Supplementary Fig. S3a). Furthermore, PBZ had no effect on splicing of *Capzb* exon 8 and *Mfn2* exon 2, which were dependent on CELF1, in *HSA*^LR^ mice^21^ (Supplementary Fig. S3b). Similarly, PBZ had no effect on splicing of *Rnf14* exon 4, which was independent on either MBNL1 or CELF1^22^, in *HSA*^LR^ mice (Supplementary Fig. S3b). Lack of the effect of PBZ on these exons suggests that PBZ specifically ameliorates aberrant splicing of MBNL1-dependent exons in *HSA*^LR^ mice. Immunofluorescence analysis showed that the immunostaining of the CLCN1 protein was markedly improved by the PBZ treatment (Fig. 3c). Thus, PBZ suppresses aberrant splicing of the *Clcn1* gene, which leads to increased expression of the CLCN1 protein on the cell membrane.

### PBZ disrupts MBNL1-CUG RNA interaction

**Figure 4:**
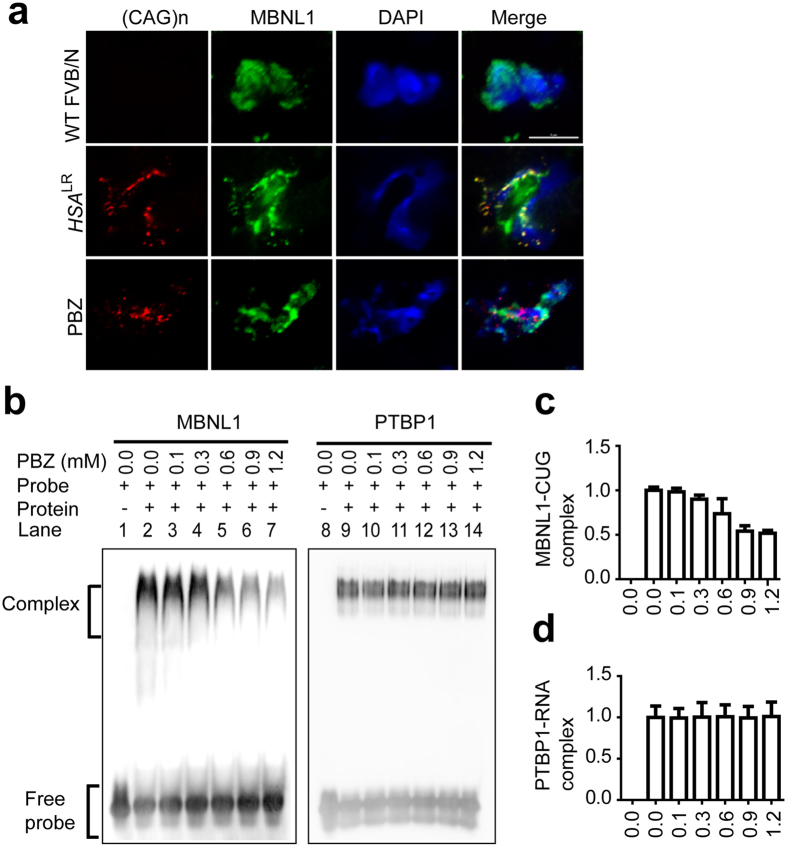
PBZ inhibits an interaction between MBNL1 and CUG RNA. (**a**) Representative RNA fluorescence *in situ* hybridization and immunohistochemistry staining of mouse quadriceps muscles to examine the effect of PBZ on the association of nuclear foci with MBNL1. The muscle sections were stained with ROX-(CAG)_n_ RNA probe (red), anti-MBNL1 antibody (green), and DAPI (blue). Scale bar = 5 μm. (**b**) EMSA analysis of binding of recombinant MBNL1 to CUG RNA (left panel), as well as of recombinant PTBP1 to polypyrimidine tract RNA (right panel), in the presence of variable concentrations of PBZ. Lanes 1, 8, only RNA; lanes 2, 9, RNA-protein complex without PBZ; lanes 3–7 and lanes 10–14, RNA–protein complex with indicated concentrations of PBZ. (**c**,**d**) The graphs show quantitative analyses of signal intensities of MBNL1-CUG complexes (**c**) and PTBP1-RNA complexes (**d**) observed in (**b**). The signal intensities are normalized to that of protein-RNA complex formed without PBZ (lane 2 or lane 9).

In DM1, MBNL1 colocalizes with CUG RNA foci. The sequestration of MBNL1 to CUG RNA foci leads to a loss-of-function of the protein^23^. To investigate whether PBZ affects association of MBNL1 with CUG RNA foci, we performed RNA fluorescence *in situ* hybridization and immunohistochemistry of skeletal muscle of *HSA*^LR^ mice (Fig. 4a). We found that MBNL1 was densely recruited to RNA foci in the quadriceps muscle in untreated *HSA*^LR^ mice (middle panels in Fig. 4a). However, colocalization of MBNL1 and RNA foci was markedly attenuated by PBZ treatment (lower panels in Fig. 4a). Quantitatively, PBZ reduced the ratio of MBNL1 foci in all RNA foci from 16.9 ± 0.5% to 4.2 ± 0.4% (mean and SD) (*p* < 0.005 by Student’s *t*-test). These results suggest that PBZ inhibits the interaction between MBNL1 and CUG RNA foci, and ameliorates sequestration of MBNL1 to the RNA foci.

To analyze whether PBZ directly inhibits MBNL1-CUG RNA interaction, we performed an electrophoretic mobility shift assay (EMSA) experiment using the recombinant MBNL1 protein and an RNA probe carrying ten copies of CUG repeats. As a control, we examined the interaction between polypyrimidine tract binding protein 1 (PTBP1) and an RNA probe carrying the polypyrimidine tract of *CHRNA1* intron 3 that we reported previously^24^. Our analysis demonstrated that PBZ suppressed formation of the MBNL1-CUG RNA complex in a dose-dependent manner (lanes 3–7 in Fig. 4b,c), whereas PBZ did not affect formation of the PTBP1-polypyrimidine tract RNA complex (lanes 10–14 in Fig. 4b,d). These results suggest that PBZ specifically inhibits the MBNL1 and CUG RNA interaction.

### PBZ suppresses methylation of CpG dinucleotides in *Mbnl1* intron 1 and promotes *Mbnl1* transcription

**Figure 5:**
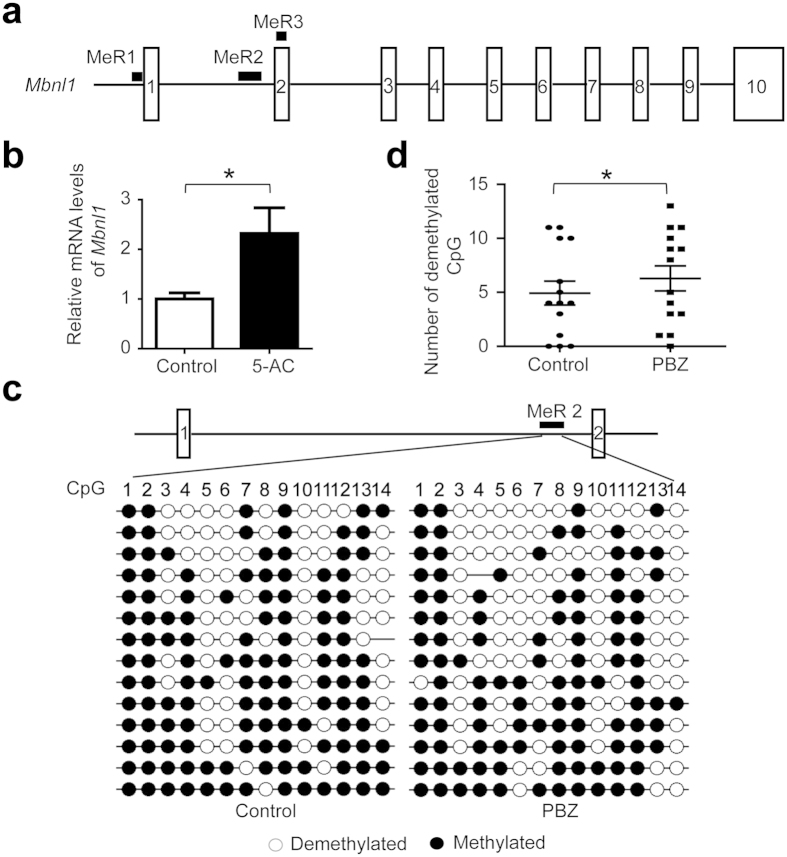
PBZ suppresses methylation of CpG dinucleotides in intron 1 of *Mbnl1*. (**a**) Schematic overview of the mouse *Mbnl1* gene structure and location of methylated regions. Exons are shown by boxes, introns by thin lines, and methylated regions (MeR1, MeR2, and MeR3) by closed squares. (**b**) DNA methylation inhibition assay by 5-AC. C2C12 cells were added with 10 μM 5-AC (5-AC) or not (control) on differentiation day 0. Total RNAs were extracted 72 h after treatment and real-time RT-PCR was performed. Expression levels of *Mbnl1* are normalized to that of *Gapdh*, and the relative mRNA expression levels are normalized to control. The mean and SD of triplicate samples are indicated. The data was analyzed by unpaired Student’s *t*-test. *p < 0.05. (**c**) Methylation analysis of MeR2. C2C12 cells were treated with 972 μM (300 ng/μl) PBZ or not (control), and DNA was extracted on differentiation day 3. Then the samples were treated with bisulfite sodium and cloned into the TA cloning vector for sequencing. For methylation analysis, 14 independent clones were sequenced for each group, and methylations of 14 CpG dinucleotides were analyzed. (**d**) The number of demethylated CpG in MeR2 was counted individually at positions 1 to 14, and is plotted for PBZ-treated (a closed square) and untreated (a closed circle) C2C12 cells. The mean and SD are indicated. *p < 0.05, by paired Student’s *t*-test.

We have demonstrated that PBZ upregulates MBNL1 expression in myogenic cells as well as in *HSA*^LR^ mice muscle. Since DNA methylation is deeply involved in the regulation of gene expression during myogenic differentiation^25^,^26^, we analyzed DNA methylation of the mouse *Mbnl1* locus. We first analyzed the effect of the 5-aza(deoxy)cytidine (5-AC), a compound known to inhibit DNA methylation, on *Mbnl1* expression. RT-PCR analysis showed that 5-AC treatment upregulated expression of *Mbnl1* mRNA in C2C12 cells (Fig. 5b), suggesting the suppressive effect of DNA methylation on *Mbnl1* expression. Next, we looked into previously published methylated DNA immunoprecipitation (MeDIP) analysis of C2C12 cells (GSE22077)^27^. We found three methylated regions (MeR) around *Mbnl1* locus in C2C12 cells: MeR1, MeR2, and MeR3 are located upstream of exon1, in intron 1, and in exon 2, respectively (Fig. 5a and Supplementary Fig. S4a). Interestingly, methylation of MeR2 is observed during myogenic differentiation as well as osteoblast differentiation, while those of MeR1 and MeR3 are observed only during osteoblast differentiation (Supplementary Fig. S4a). To examine whether PBZ affects methylation of these regions in myogenic cells, we performed bisulfite sequencing on myogenic differentiation day 3 of C2C12 cells with or without PBZ-treatment. Our analysis showed that PBZ significantly suppressed methylation of MeR2 in differentiation-induced C2C12 cells (Fig. 5c,d). Consistent with the MeDIP analysis, MeR1 was not methylated in C2C12 cells on myogenic differentiation day 3 in with PBZ-treated C2C12 cells. (Supplementary Fig. S4b).

**Figure 6:**
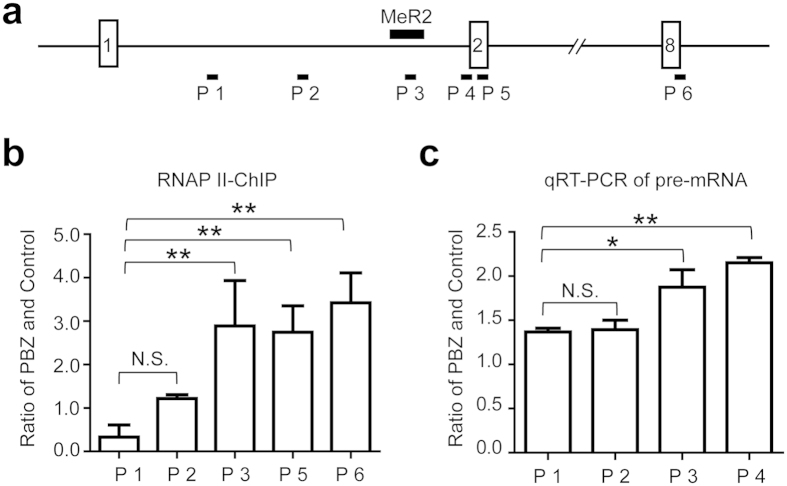
PBZ enhances *Mbnl1* transcription from the CpG dinucleotides in intron 1 of *Mbnl1*. (**a**) Quantified regions (P1 to P6) for ChIP assay and qRT-PCR analysis are shown by closed squares below the gene structure. (**b**) Real-time PCR quantification of DNA fragments precipitated with RNAP II antibody (RNAP II-ChIP). C2C12 cells were treated with or without 972 μM (300 ng/μl) PBZ for 72 h. The amplified regions are indicated in (**a**). Values are normalized to the amount of input DNA, and relative induction was calculated by diving the value of PBZ-treated cells by that of untreated cells. The mean and SD are indicated (*n* = 3). The data was analyzed by one-way ANOVA followed by Tukey’s test. **p < 0.01; N.S., not significant. (**c**) Real-time RT-PCR analysis of *Mbnl1* pre-mRNA expressions at the regions indicated in (**a**). Expression levels of *Mbnl1* are normalized to that of *Gapdh*, and the relative pre-mRNA expression level was normalized to that of untreated cells. The mean and SD are indicated (*n* = 3). The data was analyzed by one-way ANOVA followed by Tukey’s test. *p < 0.05; **p < 0.01; N.S., not significant.

Next, we examined if demethylation of MeR2 by PBZ is responsible for upregulation of *Mbnl1* mRNA. We performed chromatin immunoprecipitation (ChIP) with an antibody against RNA polymerase II (RNAP II) to see the effect of PBZ on accumulation of RNAP II in this region (Fig. 6b). We also examined the effect of PBZ on the transcription rate around MeR2 by qRT-PCR of nascent transcripts (Fig. 6c). We found that PBZ treatment increased RNAP II occupancy on (P3 in Fig. 6b) and downstream (P5 and P6 in Fig. 6b) of MeR2. Similarly, PBZ enhanced expression of nascent transcripts on (P3 in Fig. 6c) and downstream (P4 in Fig. 6c) of MeR2. These results suggest that PBZ suppresses methylation of MeR2 and enhances transcription.

*Mbnl1* in *Drosophila* has two transcriptional start sites (TSSs) at exons 1 and 2 with two distinct promoters located upstream of exons 1 and 2, respectively^28^. According to the Ensembl gene annotation release 82, *Mbnl1* in mouse has three transcriptional start sites (TSSs) at exons 1, 2, and 1′ (Supplementary Fig. S5a). We quantified mRNAs initiated at these TSSs in mouse TA muscle, and found that PBZ treatment markedly upregulated mRNA expression starting at exon 1 (Supplementary Fig. S5b). MeR2, which is in *Mbnl1* intron1, is likely to have an enhancer activity not a promoter activity on the expression of *Mbnl1*.

## Discussion

Using the drug repositioning strategy, we found that PBZ markedly elevated MBNL1 expression in myogenic cells (Fig. 1 and Supplementary Fig. S1) as well as in skeletal muscles in *HSA*^LR^ mice model (Fig. 2 and Supplementary Fig. S2). PBZ mitigated muscle pathology (Fig. 2d,e) and improved the running wheel activity and grip strength in *HSA*^LR^ mice (Fig. 2c and Supplementary Fig. S2d). Additionally, PBZ partially corrected abnormal splicing of the *Clcn1* gene and upregulated its protein expression (Fig. 3). PBZ enhanced transcription of *Mbnl1* mRNA through suppression of methylation of the CpG dinucleotides in *Mbnl1* intron 1 (Figs 5 and 6). In addition to the upregulation of MBNL1, *in cellulo* and *in vitro* analyses revealed that PBZ directly suppresses the interaction between MBNL1 and CUG RNA (Fig. 4). PBZ thus has two distinct mechanisms: one on the attenuation of sequestration of MBNL1 on CUG RNA foci, and the other on the suppression of methylation of *Mbnl1* intron 1. PBZ is accordingly able to increase availability of MBNL1 and ameliorate a loss-of-function of MBNL1 observed in DM1 pathology.

Suppression of methylation at MeR2 in *Mbnl1* intron 1 upregulated the expression of *Mbnl1* starting at the upstream exon 1 (Supplementary Fig. S5b), indicating that *Mbnl1* intron1 has an enhancer activity. Interestingly, similar enhancers located in intron 1 have been identified in other muscle-specific genes such as *Tm*^29^, *Dmd*^30^, and *Ache*^31^. Enhancers in intron 1 may have an important role for muscle-specific gene expression.

PBZ is an NSAID with anti-inflammatory, antipyretic, and analgesic activities. PBZ was approved in humans for rheumatoid arthritis and gout in 1949. Although incidental adverse effects of fatal liver disease and aplastic anemia markedly lowered the use of PBZ, PBZ is still used as an alternative drug for ankylosing spondylitis^32^,^33^. Interestingly, another NSAID, ketoprofen has been reported to suppress CUG-induced lethality in *Drosophila*^34^, and we also found that 50 μM ketoprofen upregulated the expression of *Mbnl1* mRNA 1.2-fold in C2C12 cells, which was lower than the 1.3-fold increase of *Mbnl1* mRNA by 50 μM PBZ (Supplementary Fig. S6). Ketoprofen and some other NSAIDs may have beneficial effects on a mouse model of DM1, as well as on DM1 patients.

DM1 is the most common muscular dystrophy in adults, affecting approximately one in every 8,000 individuals. However, there is as yet no curative therapy for DM1. Recent potential therapeutic approaches for DM1 have ranged from overexpression of MBNL1, RNA interference against the CUG repeats, CAG oligonucleotides, and small chemical compounds that bind to the CUG RNA and displace MBNL1 from RNA foci^9^,^35^,^36^,^37^. These novel therapeutic approaches, however, may exert unexpected toxic effects in humans. Unlike these novel therapeutic options, the optimal doses of NSAIDs including PBZ have been established and the adverse effects are predictable. In this study, we demonstrate that PBZ elevates MBNL1 expression and inhibits the MBNL1-CUG repeats interaction, which leads to amelioration of muscle weakness and muscle pathology in *HSA*^LR^ mice model. Although further analysis is required, we hope that PBZ becomes one of the rational therapeutic options for DM1 patients.

## Methods

See supplementary information for further details.

### Cell culture and drug screening

In order to screen for a drug that upregulates expression of MBNL1, C2C12 mouse myoblasts in a 12-well dish were cultured in Dulbecco’s modified Eagle’s medium (DMEM) supplemented with 10% (v/v) fetal bovine serum (FBS) containing 50 μg/mL gentamycin, and incubated at 37 °C with 5% CO_2_. When cells were grown to 70–80% confluency, 50 μM of each drug (Prestwick Chemical Library) was added. RNA was extracted at 24 h after treatment. Gene expression of *Mbnl1* was estimated by real-time RT-PCR in the LightCycler 480 (Roche Applied Science) using the SYBR Premix ExTaq (Takara Bio).

To examine the dose-responsive upregulation of MBNL1 by PBZ (P2810, LKT laboratories, Inc.) in differentiated C2C12 myotubes, C2C12 myoblasts were grown to 90% confluency in DMEM with 10% FBS, and then changed to differentiation medium containing 2% horse serum with variable concentrations of PBZ in DMEM (day 0). PBZ was dissolved in dimethyl sulfoxide (DMSO) to make a 972 mM solution, and then added to the culture medium at final concentrations of 50 to 972 μM. We also added DMSO to the medium to make the final concentration of DMSO in the medium 0.1% in all the experiments including controls without PBZ. Thus, the effect of PBZ in cultured cells was analyzed in the presence of 0.1% DMSO and was always compared to control cells cultured in 0.1% DMSO. Cells were serially harvested on days 1, 3, and 5, and total RNA was extracted with the TRIzol reagent (Thermo Fisher Scientific). Gene expression of *Mbnl1* was estimated as stated above.

### Administration of PBZ to DM1 mouse model

All mouse studies were approved by the Animal Care and Use Committee of the Nagoya University Graduate School of Medicine, and were performed in accordance with the relevant guidelines. *HSA*^LR^ transgenic mice that express the human α-skeletal actin mRNA containing (CUG)_250_ in the 3′ UTR were kindly provided by Dr. Charles A. Thornton at University of Rochester^18^. Mice were divided into three groups (*n* = 5 in each group): (i) untreated *HSA*^LR^ mice, (ii) PBZ-treated *HSA*^LR^ mice, and (iii) untreated wild-type FVB/N mice. In Supplementary Figure S3, mice were divided into two groups (*n* = 3 in each group): (i) untreated wild-type FVB/N mice and (ii) PBZ-treated wild-type FVB/N mice. At eight weeks after birth, *HSA*^LR^ mice started to take 16.7 mg/kg/day of PBZ, which is about three times more than that taken by patients as an NSAID (300 mg/day). Body weights were measured every week and the calculated amount of PBZ was directly dissolved in drinking water assuming that each mouse drank 3–5 mL of water every day. Mice were sacrificed twelve weeks after initiation of treatment.

### Running wheel test

Each mouse was kept in an individual cage with a counter-equipped running wheel (diameter = 14.7 cm, width = 5.2 cm; Ohara Medical) for 48 h to record the number of wheel rotations. The counter sensed rotations in both directions. Each mouse was moved to the running wheel cage every 14 days. We measured voluntary activities from 4 to 8 weeks of age without treatment, and from 8 to 20 weeks of age with PBZ treatment.

### Grip strength test

The peak grip force was measured using a grip strength meter (Bioseb - *In Vivo* Research Instruments), according to the manufacturer’s protocol. Mice were allowed to grasp a wire mesh with their forelimbs and hindlimbs, and then pulled steadily by their tails horizontally until they lost their grip. Measurements were performed 3 times using untreated wild-type FVB/N mice, untreated *HSA*^LR^ mice, and PBZ-treated *HSA*^LR^ mice^38^. PBZ treatment was performed as described above.

### Hematoxylin and eosin staining and immunostaining of quadriceps muscles

Quadriceps muscles of three group mouse were snap-frozen in isopentane chilled with liquid nitrogen. Quadriceps muscles were sliced at 10 μm with a cryostat. Hematoxylin and eosin (H&E) staining was done according to the standard procedures.

Frozen sections (10 μm) of quadriceps muscle were permeabilized with acetone on ice for 10 min and dried. Then, the sections were blocked in 5% horse serum for 1 h at 4 °C, rinsed with phosphate-buffered saline (PBS) for 5 min three times. The sections were immunostained with an antibody against chloride channel-1 (1:50, CLC11-A, Alpha Diagnostic International) and then with anti-rat FITC (1:100, SAB1038, Open Biosystems). Nuclei were visualized by staining with diamidino-2-phenylindole (DAPI, Vector Laboratories).

### Splicing analysis and quantitative RT-PCR

Total RNA was extracted from mouse TA muscles and quadriceps muscles at 12 weeks after initiation of PBZ treatment using the RNeasy mini kit (Qiagen) according to the manufacturer’s instructions. cDNA was synthesized using a random primer (Thermo Fisher Scientific) and ReverTraAce (Toyobo), and PCR amplifications were performed using GoTaq (Promega) for 30–35 cycles. The primer sequences used for RT-PCR are shown in Supplementary Table S2. The intensities of RT-PCR-amplified spliced products were quantified with the ImageJ 1.42q software (http://imagej.nih.gov/ij/). We then estimated the ratio of exon inclusion by dividing the signal intensity of the upper band by the sum of signal intensities of two bands.

Real-time RT-PCR was performed with the LightCycler 480 (Roche Life Science) using the SYBR Premix ExTaq (Takara Bio). *Gapdh* and *Actb* (β-actin) mRNAs were used as the housekeeping genes. We then normalized the expression level of a specific gene by that of the housekeeping gene. PCR primers are shown in Supplementary Tables S3 and S6. All real-time RT-PCR experiments were performed in three independent experiments.

### *In situ* hybridization of CUG-repeat RNA combined with MBNL1 immunostaining

*In situ* hybridization was performed essentially as described elsewhere^6^,^39^. Briefly, frozen sections (6 μm) of quadriceps muscles were dried for 30 min, fixed in 2% paraformaldehyde in PBS for 10 min at room temperature, washed in PBS three times for 5 min, permeabilized in 2% acetone in PBS (pre-chilled at 4 °C) for 5 min, and soaked in 30% formamide and 2 × SSC at room temperature for 10 min. The sections were hybridized with a probe (1 ng/μl) for 2 h at 37 °C in a binding buffer [30% formamide, 2 × SSC, 0.02% bovine serum albumin (BSA), 66 μg/ml yeast tRNA (Sigma), 2 mM Ribonucleoside Vanadyl Complex (Sigma)], and then washed for 30 min in 2 × SSC including 30% formamide at 42 °C followed by washing with 1 × SSC for 30 min at room temperature. Following the post-hybridization wash with 1 × SSC, the sections were incubated overnight at 4 °C with rabbit anti-MBNL1 antibody (1:1000, A2764), which was a kind gift from Dr. Charles A. Thornton at University of Rochester. The sections were washed three times with PBS for 2 min, incubated with anti-rabbit FITC (1:200, FI-1000, Vector Laboratories) for 30 min at room temperature, and washed five times in PBS. The sections were stained with 33 nM DAPI. A probe comprised of six copies of CAG repeats was synthesized by Hokkaido System Science (HPLC-purified 2-O-methyl RNA 20-mers), and was labeled with Rox at the 5′-end.

The number of nuclear foci was counted in six randomly selected visual fields of a section of quadriceps muscle of six mice. Association of MBNL1 with nuclear foci was automatically analyzed with the IN Cell Analyzer 6000 Cell Imaging system (GE Healthcare Life Sciences).

### EMSA assays

The RNA probes with biotin labeling at the 5′-end were obtained from Hokkaido System Science. The sequences of RNA probes used in this study were: CUG repeat RNA probe, 5′-GCCUGCUGCUGCUGCUGCUGCUGCUGCUGCUGGC-3′ simulating abnormally expanding 3′ UTR of *DMPK*; polypyrimidine tract RNA probe, 5′-UUUCUCCUUUUCUGUGGGUGGACAGGGUGACAUGGUA-3′ arising from *CHRNA1*^24^. Recombinant MBNL1 protein (6 μg) and GST-PTBP1 protein (6 μg) were incubated with the RNA probe (2.5 pmol/μl) for 30 min at room temperature in 20 μl of binding buffer (10 mM Tris-HCl pH 7.5, 50 mM KCl, and 1 mM DTT). Then, variable concentrations of PBZ were added in the reaction mixture for 15 min at room temperature, and the samples were subjected to nondenaturing polyacrylamide gel electrophoresis. The samples were transferred onto a positively charged nylon membrane (11209299001, Roche Life Technologies), and UV-crosslinked at 1200 μJ/cm^2^. The signals of gel-shifted RNA probe were detected by the streptavidin-horseradish peroxidase conjugate (RPN1231-2 ML, GE Healthcare Life Sciences) using the Image Quant LAS 4000 mini (GE Healthcare Life Sciences).

### Chromatin immunoprecipitation

C2C12 cells were treated with 972 μM (300 ng/μl) PBZ on differentiation day 0, and incubated for 72 h. Then, ChIP assays were performed as described previously^40^. Briefly, cells were cross-linked by 1% formaldehyde in DMEM for 10 min at room temperature. Cell lysates were sonicated until chromatin fragments became 200–1000 bp in size. Anti-mouse RNAP II (SC-899, N-20, Santa Cruz Biotechnology) monoclonal antibody was used for DNA immunoprecipitation (IP-DNA). After immunoprecipitation, recovered chromatin samples were subject to real-time PCR. The primer pairs for ChIP assay are shown in Supplementary Table S7.

### Statistical analyses

All data are presented as the mean and SD. Statistical difference was estimated either by Student’s *t*-test, one-way ANOVA followed by Tukey’s post-hoc test, or two-way repeated measures ANOVA followed by Bonferroni’s post-hoc test.

## Additional Information

**How to cite this article**: Chen, G. *et al*. Phenylbutazone induces expression of MBNL1 and suppresses formation of MBNL1-CUG RNA foci in a mouse model of myotonic dystrophy. *Sci. Rep*. **6**, 25317; doi: 10.1038/srep25317 (2016).

## Supplementary Material

Supplementary Information
