# Supplementary material for: Phenylbutazone induces expression of MBNL1 and suppresses formation of MBNL1-CUG RNA foci in a mouse model of myotonic dystrophy
Source: Sci Rep. 2016 Apr 29;6:25317. doi: 10.1038/srep25317 (PMC4850456; doi:10.1038/srep25317)
Supplement: Supplementary Information [file srep25317-s1.pdf]

Phenylbutazone induces expression of MBNL1 and suppresses formation of MBNL1-CUG RNA foci in a mouse model of myotonic dystrophy

Guiying Chen[1], Akio Masuda[1,*], Hiroyuki Konishi[2], Bisei Ohkawara[1], Mikako Ito[1], Masanobu Kinoshita[3], Hiroshi Kiyama[2], Tohru Matsuura[1,4], Kinji Ohno[1]

[1]Division of Neurogenetics, Center for Neurological Diseases and Cancer, Nagoya University Graduate School of Medicine, Nagoya, Japan
[2]Division of Functional Anatomy and Neuroscience, Nagoya University Graduate School of Medicine, Nagoya, Japan
[3]Department of Frontier Health Sciences, Graduate School of Human Health Sciences, Tokyo Metropolitan University, Tokyo, Japan
[4]Division of Neurology, Department of Medicine, Jichi Medical University, Shimotsuke, Japan

*****Address correspondence to: Akio Masuda, Division of Neurogenetics, Center for Neurological Diseases and Cancer, Nagoya University Graduate School of Medicine, 65 Tsurumai, Showa-ku, Nagoya, Aichi 466-8550, Japan
Phone: +81-52-744-2447, Fax: +81-52-744-2449, e-mail: amasuda@med.nagoya-u.ac.jp

Supplementary information includes:

Supplementary methods.
Supplementary Tables S1-S7.
Supplementary Figures S1-S6.

Supplementary methods

Western blot analyses.

Mouse TA muscles and quadriceps muscles were immediately frozen in liquid nitrogen and ground with a mortar and pestle. The powdered tissues were solubilized in PLC lysis buffer [50 mM HEPES pH 7.0, 150 mM NaCl, 10% (vol/vol) glycerol, 1% (vol/vol) TritonX-100, 1.5 mM $MgCl_2$, 1 mM EGTA, 100 mM NaF, 10 mM NaPPi (sodium pyrophosphate)] with protease inhibitors (1 µg/µl aprotinin, 1 µg/µl leupeptin, 1 mM PMSF) and gently sonicated two times for 2 min.

C2C12 cells were lysed with PLC lysis buffer with protease inhibitors, and whole cell lysates were extracted as described above. The lysates were incubated for 30 min on ice, then cleared after centrifugation for 30 min at 15,400 × *g* at 4˚C. The cleared lysates were resolved with SDS/PAGE and blotted on to a PVDF membrane, as previously described[1]. The membrane was stained with anti-MBNL1 mouse monoclonal antibody (1:200, 4A8, sc-136165, Santa Cruz) and anti-CELF1 mouse monoclonal antibody (1:200, 3B1, sc-20003, Santa Cruz), and re-blotted with anti-GAPDH rabbit polyclonal antibody (1:5000, G9545, Sigma-Aldrich).

Expression and purification of recombinant proteins.

The bacterial expression vector, GST-MBNL1, carrying GST cDNA fused to MBNL1 cDNA (amino acids 1-260) was kindly provided by Dr. Andrew Berglund at University of Oregon[2]. Another bacterial expression vector, GST-PTBP1, carrying GST cDNA fused to PTBP1 cDNA was constructed by inserting a previously cloned PTBP1 cDNA (BC013684)[3] into the GST-fusion vector pGEX-6P-1 (GE Healthcare Life Sciences) at EcoRI and XhoI sites. Protein expression was induced using BL21 Star (DE3)pLysS One Shot Chemically Competent *E. coli* (Thermo Fisher Scientific) with 0.25 mM IPTG (Sigma) at an OD600 ≈ 0.5-1.0, for 4 h at 37˚C. Cells that express MBNL1 were lysed in 10 ml of buffer (100 mM NaCl, 50 mM Tris-HCl pH 8.5, 1 mM EDTA, and 1 mM DTT). Similarly, cells that expressed PTBP1 were lysed in 10 ml of buffer (500 mM NaCl, 25 mM Tris-HCl pH 7.5, 1 mM β-mercaptoethanol, and 5% glycerol) using 1 mg/mL of lysozyme (Sigma) followed by sonication (3 × 30 s). The cell extracts were centrifuged for 15 min at 21,160 × *g*, and the supernatants were collected. GST-fusion protein was bound to Glutathione-Sepharose 4B beads (GE Healthcare Life sciences) for 45 min at 4˚C, and the beads were washed 3 times with cleavage buffer (100 mM NaCl, 50 mM Tris-HCl pH 8.0, 1 mM EDTA, and 1 mM DTT). GST-MBNL1 was cleaved from the affinity tag with PreScission Protease (GE Healthcare Life Sciences) according to the manufacturer’s instructions. GST-PTBP1 was eluted in elusion buffer (50 mM Tris-HCl pH 8.5, 100 mM NaCl, 1 mM DTT, 1 mM EDTA and 30 mM Glutathione Reduced Form). The supernatants containing recombinant proteins were collected from the beads, and stored at -80˚C.

DNA methylation-inhibition assay.

C2C12 cells were seeded in a 60-mm culture dish. After confluency, the culture medium was replaced with the differentiation medium containing 2% horse serum. Cells were treated with 10 µM 5-AC (A2385, Sigma) on differentiation day 0. RNA was extracted 72 h after treatment. Expressions of *Mbnl1* and *Gapdh* mRNAs were quantified by real-time RT-PCR with primers shown in Supplementary Table S3.

DNA extraction and CpG methylation analysis by bisulfite-sequencing PCR.

Genomic DNA was extracted from C2C12 cells using QIAamp DNA Mini Kit (Qiagen). Non-methylated cytidines in DNA (2.5 µg) were converted to uridines with MethylEasy^{TM} Xceed Rapid DNA Bisulfite modification kit (Cat # ME002, Human Genetic Signatures) according to the manufacturer's instructions. Pairs of bisulfite-sequencing PCR (BSP) primers were used to amplify the methylated MeR1 and MeR2 regions of *Mbnl1* (Supplementary Fig. S3). Primer sequences are shown in Supplementary Table S4 and S5. The PCR products were run on an agarose gel followed by purification with the Wizard SV Gel and PCR Clean-Up System (Promega), and subcloned into the TA cloning vector (pGEM-T Easy Vector, Promega). To identify the methylated CpG dinucleotides, we sequenced 14 independent clones for each sample with the CEQ 2000 DNA Analysis System (Beckman Coulter). The methylation pattern was analyzed with the QUMA (Quantitative Tool for Methylation Analysis) software (http://quma.cdb.riken.jp/) using the default parameters[4].

Quantitative RT-PCR.

To quantify *Mbnl1* mRNAs initiated at different TSSs (1a, 1b, 2, and 1' in Supplementary Fig. S5a), we performed real-time RT-PCR using primers shown in Supplementary Table S6. *Mbnl1* transcripts 1a and 1b were quantified using a primer pair E/1F- E/2R and E1/F-E3/R, respectively. Whole *Mbnl1* transcripts were quantified with a primer pair E3/F-E4/R. Then, the expression level of residual *Mbnl1* transcripts (2+1') was estimated by subtracting expression level of 1a and 1b from that of whole *Mbnl1* transcripts.

Liver function tests.

Liver function was evaluated with serum levels of total protein (TP), alanine aminotransferase (ALT), aspartate aminotransferase (AST), alkaline phosphatase (ALP), lactate dehydrogenase (LDH), leucine aminopeptidase (LAP), γ-glutamyltransferase (r-GT), Cholinesterase (ChE), and total bilirubin levels (T-BIL). These parameters were all analyzed at Oriental Yeast Co., Ltd. (Japan).

Supplementary references

1. Masuda, A. et al. CUGBP1 and MBNL1 preferentially bind to 3' UTRs and facilitate mRNA decay. *Sci Rep* **2**, 209 (2012).
2. Warf, M.B., Nakamori, M., Matthys, C.M., Thornton, C.A. & Berglund, J.A. Pentamidine reverses the splicing defects associated with myotonic dystrophy. *Proc Natl Acad Sci U S A* **106**, 18551-6 (2009).
3. Bian, Y. et al. Tannic acid facilitates expression of the polypyrimidine tract binding protein and alleviates deleterious inclusion of CHRNA1 exon P3A due to an hnRNP H-disrupting mutation in congenital myasthenic syndrome. *Hum Mol Genet* **18**, 1229-37 (2009).
4. Kumaki, Y., Oda, M. & Okano, M. QUMA: quantification tool for methylation analysis. *Nucleic Acids Res* **36**, W170-5 (2008).
5. Hupkes, M. et al. DNA methylation restricts spontaneous multi-lineage differentiation of mesenchymal progenitor cells, but is stable during growth factor-induced terminal differentiation. *Biochim Biophys Acta*

1813, 839-49 (2011).

Supplementary Table S1. Serum levels of liver function parameters

Mouse	TP (g/dL)	AST (IU/L)	ALT (IU/L)	ALP (IU/L)	LDH (IU/L)	LAP (IU/L)	γ-GT (IU/L)	ChE (IU/L)	T-BIL (mg/dL)
WT FVB/N-1	5.3	66	24	270	844	46	3 >	30	0.04
WT FVB/N-2	5.6	144	68	354	1396	44	6 >	28	0.02
WT FVB/N-PBZ-1	5.3	75	27	263	1046	42	3 >	28	0.03
WT FVB/N-PBZ-2	5.6	81	21	252	1420	33	3 >	29	0.04
HSA^{LR}-1	7.3	250	30 >	216	3620	30	3 >	28	0.21
HSA^{LR}-2	5.3	123	33	243	1890	37	3 >	24	0.06
HSA^{LR}-PBZ-1	5.7	114	60	241	1135	46	3 >	28	0.06
HSA^{LR}-PBZ-2	5.3	90	39	253	1340	33	3 >	28	0.04

Mice were divided into four groups (n = 2 in each group): untreated wild-type FVB/N mice (WT FVB/N), PBZ-treated wild-type FVB/N mice (WT FVB/N-PBZ), untreated *HSA*^{LR} mice (*HSA*^{LR}), and PBZ-treated *HSA*^{LR} mice (PBZ). Mice were sacrificed twelve weeks after initiation of treatment, and serum was harvested. Liver function was evaluated with serum levels of total protein (TP), alanine aminotransferase (ALT), aspartate aminotransferase (AST) and alkaline phosphatase (ALP), lactate dehydrogenase (LDH), leucine aminopeptidase (LAP), γ-glutamyltransferase (r-GT), Cholinesterase (ChE), and total bilirubin levels (T-BIL). These parameters indicate that PBZ given to wild-type FVB/N mice and *HSA*^{LR} mice had no adverse effect on the liver function.

Supplementary Table S2. Primer sequences for RT-PCR

Gene	Forward	Reverse
Clcn1	GTCCTCAGCAAGTTTATGTCC	GAATCCTCGCCAGTAATTCC
Nfix	TCGACGACAGTGAGATGGAG	CAAACTCCTTCAGCGAGTCC
Rpn2	CCCTCAAGTACCTGGCTGTC	CATGAACTGTCCCCACTCCT
Capbz	GCACGCTGAATGAGATCTACTTTG	CCGGTTAGCGTGAAGCAGAG
Mfn2	AGCCATGTCCACGATGCCCA	ATGTAGGCCCCCAGCTGCTCAA
Rnf14	AATGTCGGCAGAAGACCTGG	CCCACAGGTTGTCTAGGTGC
Gapdh	ACCACAGTCCATGCCATCAC	TCCACCACCCTGTTGCTGTA

Supplementary Table S3. Primer sequences for real-time RT-PCR

Gene	Forward	Reverse
Mbnl1	GTTGCAGCCCGTGCCAATGTT	TTGGGGAAACAGGCCCCAGGTAA
Gapdh	ACCCCTTCATTGACCTCAAC	TCCCGTTGATGACAAGCTTC

Supplementary Table S4. Primer sequences for bisulfite sequencing of MeR2

	Forward	Reverse
PCR segment 1	ATTTATGTTTTTGGGATAGTTTTTT	CAAAAAAAATAACACATCTCTATTC
PCR segment 2	AGATGTGTTATTTTTTTTGGTTTTT	TATATATTTTTAATTTATTTTCCTTTTAAA[a]
Seq. Primer 1F	GAAGAATTGGTTTTATTTTGATAG	
Seq. Primer 1R		ATAAATAACCAATTTATAATTTTAAAAATT
Seq. Primer 2R		TATATATTTTTAATTTATTTTCCTTTTAAA[a]
Seq. Primer 3R		TATCAAAATAAAACCAATTCTTCCC

The upstream and downstream segments of MeR2 were individually amplified. PCR segment 1 was sequenced with Seq. Primers 1F and 1R. PCR segment 2 was sequenced with Seq. Primers 2R and 3R. [a]Note that the reversed primer for PCR segment 2 and Seq. Primer 2R are identical.

Supplementary Table S5. Primer sequences for bisulfite sequencing of MeR1

	Forward	Reverse
Me primer	TATGTTTTCGTGATTCGGATTC	GTAACCGTACAAACTACCCG
Un-Me primer	GTTATGTTTTTGTGATTTGGATTTG	CACATAACCATACAAACTACCCAC

Supplementary Table S6. Primer sequences for *Mbnl1* isoform analysis

Primer	Forward	Reverse
E1/F	GGAGTCGCGATCCCACAAT	
E2/R		GCAAGCAAGGCAAAAGCAGA
E3/F, R	GTTGCAGCCCGTGCCAATGTT	GGCTAACTGCATTTGCTGGG
E4/R		TTGGGGAAACAGGCCCCAGGTAA

Supplementary Table S7. Primer sequences for ChIP assay and amplification of pre-mRNA

Position[a]	Forward	Reverse
P 1	CAATGGTGACCGTGCAAGTG	AACTCCTGATGCAGTGGTCC
P 2	TGGCTGACGTAAGTGGAGCA	TACCTCAATCCCCACTGCGA
P 3	CCCCTGGAAACGTGGTTTAG	GGTCACCACCTCTTCAGTCC
P 4	GGTTGTGCTGCTATAGGACATCAG	GTCAGTCTAGCTGGCAGCAAGC
P 5	CCGATTGCACCACCAAACTC	ATGAAAACGTGGCAGACCCT
P 6	ACATCTGCCACAAGTGTTCCC	CAGAATGGGAGTTCCATGAGC

[a]Positions are indicated in Fig. 6.

Legends for Supplementary Figures

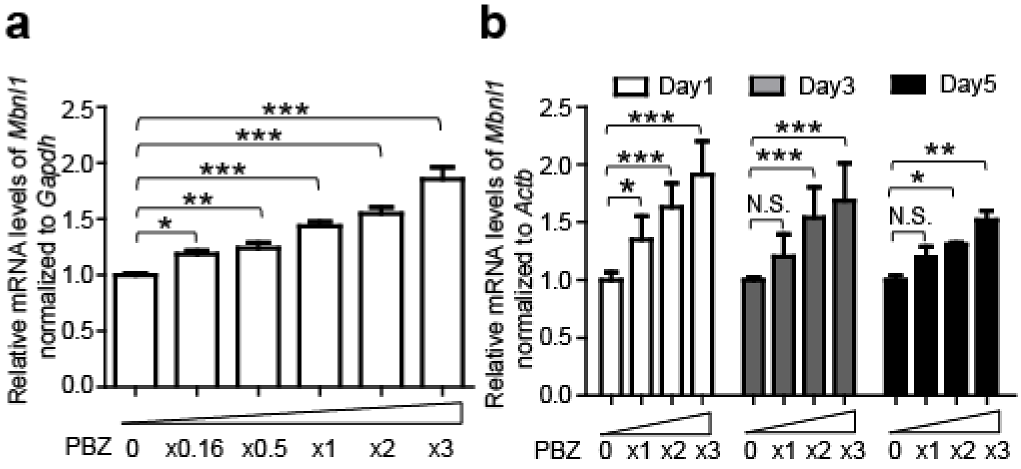

Supplementary Figure S1. Real-time RT-PCR to estimate expression of *Mbnl1* in undifferentiated (a) and differentiating (b) C2C12 cells. (a) Cells were treated with variable concentrations of PBZ for 24 h. Expression levels of *Mbnl1* are normalized to that of *Gapdh* and the relative mRNA expression levels are normalized to that of untreated cells. The data are analyzed by one-way ANOVA followed by Tukey’s test. **(b)** Cells were treated with variable concentrations of PBZ from differentiation day 0. Expression levels of *Mbnl1* are normalized to that of *Actb*, instead of *Gapdh* in Figure 1a, and the relative mRNA expression levels are normalized to that of untreated cells on the same differentiation day. The relative expression levels of *Mbnl1* in untreated cells normalize for that on day 1 were 1.0 ± 0.07 on day 1, 1.17 ± 0.03 on day 3, and 1.45 ± 0.05 on day 5 (mean and SD, $n = 3$). The data are analyzed by two-way repeated measures ANOVA followed by Bonferroni post-hoc test. (**a** and **b**) The mean and SD of three independent experiments are indicated. Concentrations of PBZ: 0, 0 µM; x 0.16, 50µM (15 ng/µl); 0.5x, 162µM (50 ng/µl); x1, 324 µM (100 ng/µl); x2, 648 µM (200 ng/µl); and x3, 972 µM (300 ng/µl). *$p < 0.05$; **$p < 0.01$; ***$p < 0.001$; N.S., not significant.

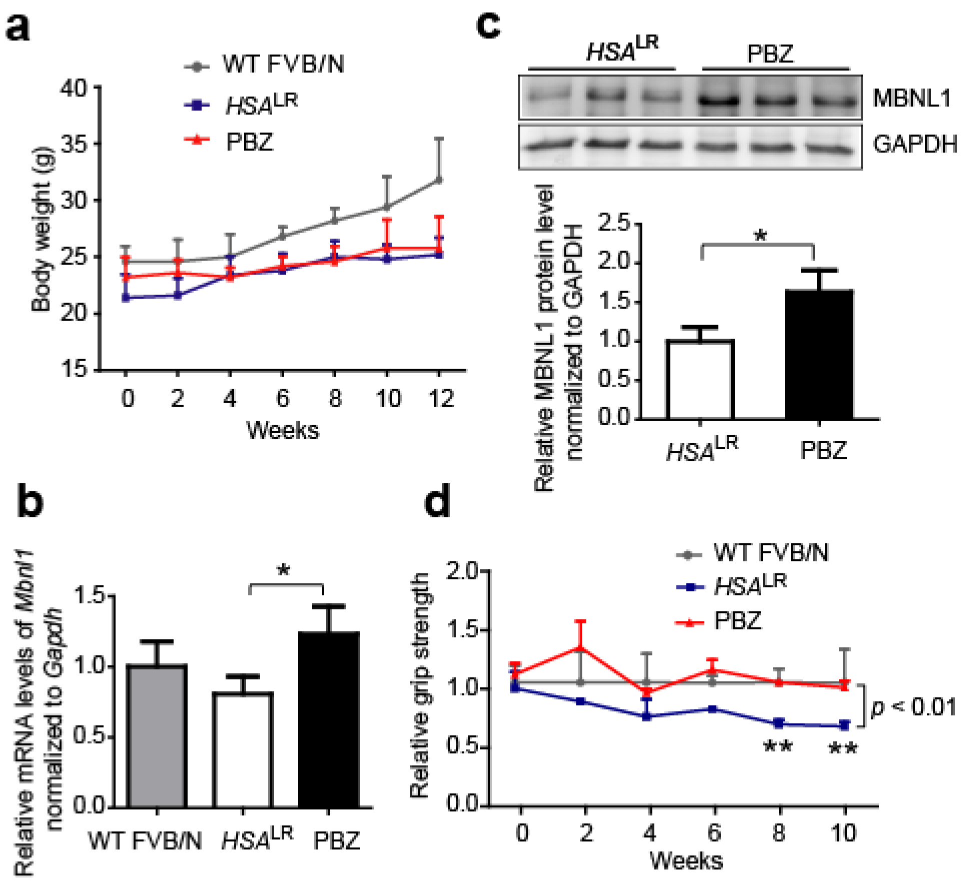

Supplementary Figure S2. PBZ upregulates MBNL1 expression in quadriceps muscle, and improves muscle weakness. (**a**) Body weights of PBZ-treated *HSA*^{LR} mice. Mouse body weights were not affected by PBZ treatment. Body weights of untreated wild-type FVB/N mice (WT FVB/N), untreated *HSA*^{LR} mice (*HSA*^{LR}), and PBZ-treated *HSA*^{LR} mice (PBZ) were measured every other week ($n = 5$). $p = 0.12$ between *HSA*^{LR} and PBZ by two-way repeated measures ANOVA. (**b**) Real-time RT-PCR analysis to estimate expression of *Mbnl1* in quadriceps muscle. Expression levels of *Mbnl1* are normalized to the level of *Gapdh* and the relative mRNA expression levels are normalized to WT FVB/N. The mean and SD of five mice in each group are indicated. The data were analyzed by one-way ANOVA followed by Tukey's test. *$p < 0.05$. (**c**) Western blotting analysis of MBNL1 in mouse quadriceps muscle. Whole protein was extracted from quadriceps muscle. Expression levels of MBNL1 are normalized to the level of GAPDH and the relative expression levels are normalized to *HSA*^{LR}. The mean and SD of three mice in each group are indicated. The data were analyzed by Student's *t*-test. *$p < 0.05$. (**d**) Grip strength analysis of untreated wild-type FVB/N mice (WT FVB/N), untreated *HSA*^{LR} mice (*HSA*^{LR}), and PBZ-treated *HSA*^{LR} mice (PBZ). Grip strengths of untreated *HSA*^{LR} mice and PBZ-treated *HSA*^{LR} mice are normalized to that of WT-FVB/N mouse. Plots show mean and SD of the relative grip strength of three mice. The data were analyzed using two-way repeated measures ANOVA followed by Bonferroni post-hoc test. **$p < 0.01$, compared to untreated WT FVB/N mice.

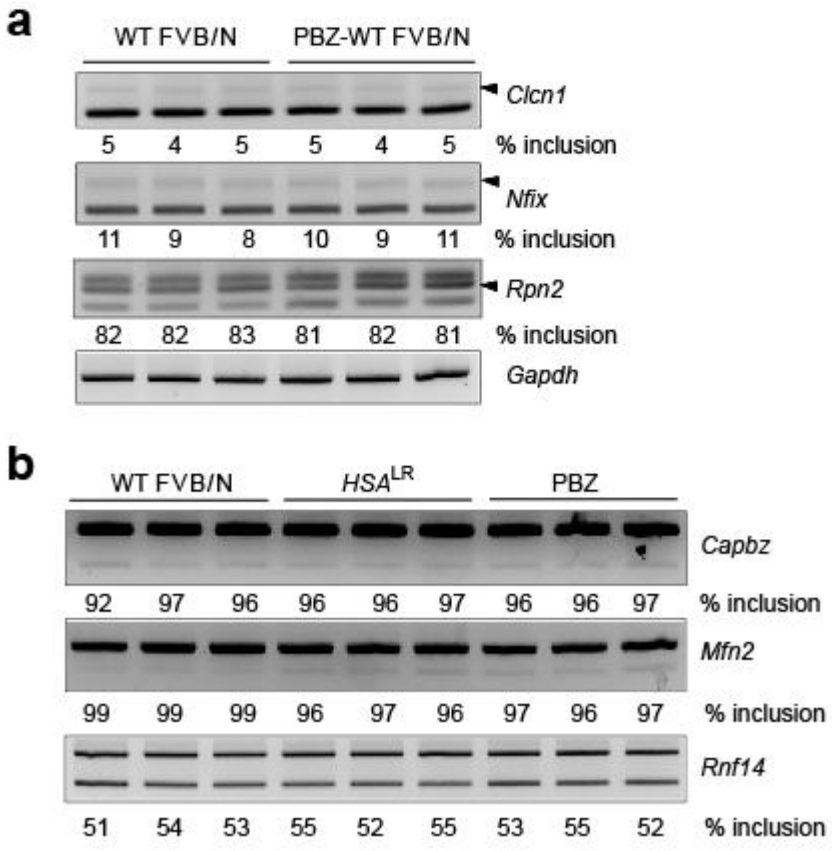

Supplementary Figure S3. PBZ has no effect on splicing of exons in wild-type FVB/N mice, which are aberrantly included in *HSA*^{LR} mice, and on splicing of MBNL1-independent exons in *HSA*^{LR} mice. (**a**) RT-PCR analysis of splicing of *Clcn1* exon 7a, *Nfix* exon 7, and *Rpn2* exon 17 in TA muscles of untreated (WT FVB/N) and PBZ-treated (PBZ-WT FVB/N) wild-type FVB/N mice. Arrowheads point to abnormal fragments increased in *HSA*^{LR} mice. (**b**) RT-PCR analysis of splicing of *Capzb* exon 8 and *Mfn2* exon 2 (dependent of CELF1) and of *Rnf14* exon 4 (independent of MBNL1 or CELF1) in TA muscles of wild-type FVB/N mice (WT FVB/N), untreated *HSA*^{LR} mice (*HSA*^{LR}), and PBZ-treated *HSA*^{LR} mice (PBZ). The numbers below each lane represent the percentage of exon inclusion. The ratio of inclusion of an exon was calculated as explained in Methods.

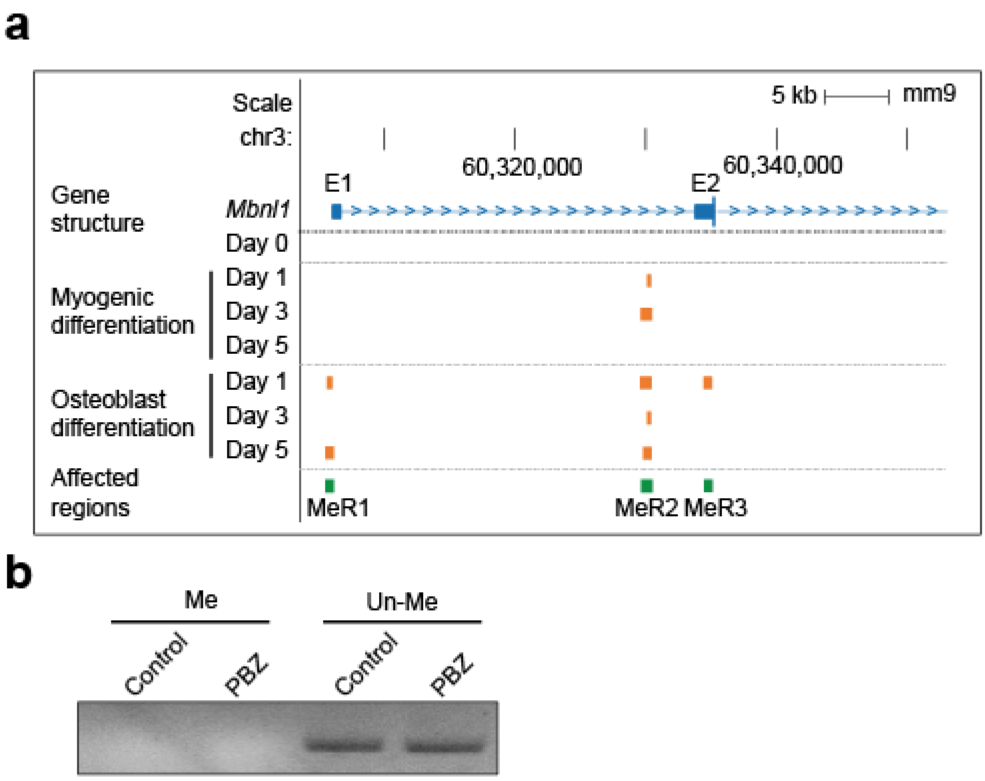

Supplementary Figure S4. Three methylated regions detected in MeDIP-seq analysis of C2C12 cells (GSE22077) in the *Mbnl1* genomic region (GRCm37/mm9, chr3: 60,304,792 to 60,368,300). (**a**) Exon 1 (E1) and exon 2 (E2) are indicated by blue boxes, and introns by blue thin lines. Methylated regions, which were detected in the MeDIP-seq analysis of C2C12 cells during myogenic differentiation or osteoblast differentiation (GSE22077)[5], are indicated by orange boxes. We observe that three regions (MeR1, MeR2 and MeR3) are methylated in the course of the differentiation. MeR1, positions 60,304,792 to 60,305,159; MeR2, positions 60,330,708 to 60,331,205; and MeR3, positions 60,333,037 to 60,333,293 on chromosome 3 according NC_000069.6 (GRCm37/mm9). (**b**) Methylation-specific PCR analysis of MeR1 region using pairs of primers specific for methylated (Me) and un-methylated (Un-Me) DNA.

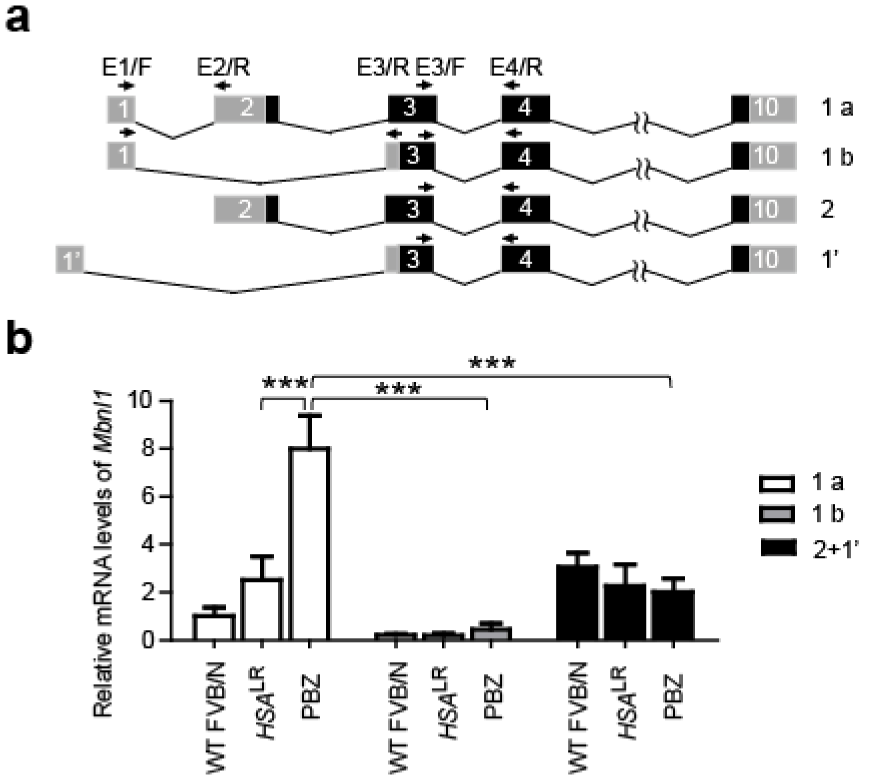

Supplementary Figure S5. Expression of *Mbnl1* mRNAs transcribed from different start sites in skeletal muscle. (**a**) TSS variation in *Mbnl1* mRNA. According to the Ensembl annotation, four different transcripts start from exon 1 (1a and 1b), exon 2 (2) and exon 1' (1'). Exons are shown by boxes, coding regions by closed boxes, and untranslated regions by shaded boxes. Arrows indicate locations of PCR-primers to quantify each alternative *Mbnl1* transcript. The number of transcripts 2+1' was estimated by subtracting transcripts 1a and 1b from the whole transcripts measured by E3/F and E4/R. (**b**) Real-time RT-PCR analysis of *Mbnl1* mRNA expressions with different TSSs. Total RNAs were extracted from skeletal muscles of untreated wild-type FVB/N mice (WT FVB/N), untreated HSA^{LR} mice (HSA^{LR}), and PBZ-treated HSA^{LR} mice (PBZ). Real-time RT-PCR analysis was performed using the primers shown in (**a**). Expression level of each transcript, which is calculated as in Methods, is normalized to that of *Gapdh*, and the relative mRNA expression levels are further normalized to that of transcript (1a) of WT FVB/N mice. The mean and SD of five mice in each group are indicated. The data were analyzed by two-way ANOVA followed by Bonferroni post-hoc test. ***$p < 0.001$.

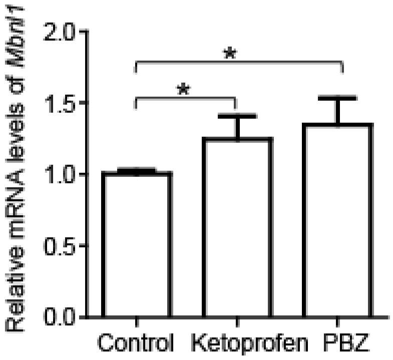

Supplementary Figure S6. The effect of 50 µM ketoprofen and 50 µM PBZ on the expression of *Mbnl1* mRNA in C2C12 myoblasts. Cells were added with either Ketoprofen or PBZ at 70% confluency. The cells were harvested 24 h after addition of either drug. The amount of *Mbnl1* mRNA was quantified by qRT-PCR, and was normalized to that of untreated cells. The mean and SD are indicated ($n = 3$). *$p < 0.05$, by unpaired Student's *t*-test.
